# Health-related quality of life in overweight and obese youths: Results of a multicenter study

**DOI:** 10.1186/1477-7525-8-36

**Published:** 2010-04-07

**Authors:** Nora Wille, Monika Bullinger, Reinhard Holl, Ulrike Hoffmeister, Reinhard Mann, Cornelia Goldapp, Thomas Reinehr, Joachim Westenhöfer, Andreas Egmond-Froehlich, Ulrike Ravens-Sieberer

**Affiliations:** 1 Research Section Child Public Health, Dept. of Psychosomatics in Children and Adolescents, University Clinic Hamburg-Eppendorf, Martinistr. 52, 20246 Hamburg, Germany; 2 Dept for Medical Psychology, University Clinic Hamburg-Eppendorf, Martinistr. 52, 20246 Hamburg, Germany; 3 Dept for Epidemiology, University of Ulm, Albert-Einstein-Allee 47, 89081 Ulm, Germany; 4 Federal Centre for Health Education (BZgA), Ostmerheimer Str. 220, 51109 Köln, Germany; 5 Dept for Paediatric Nutrition Medicine, Vestische Hospital for Children and Adolescents Datteln, University of Witten/Herdecke, Dr F. Steiner Str. 5, 45711 Datteln, Germany; 6 Dept for Health Sciences, University of Applied Sciences, Lohbruegger Kirchstrasse 65, 21033 Hamburg, Germany; 7 Children's Rehabilitation Clinic Schönsicht, Oberkälberstein 1-11, Kälbersteinstrasse 14, 83471 Berchtesgaden, Germany

## Abstract

**Background:**

We examined treatment-seeking overweight and obese youths to better understand the gender, age, and treatment modality differences in generic and disease-specific health-related quality of life (HRQOL).

**Methods:**

This multicenter study included 1,916 patients (mean = 12.6 years; 57% females; mean zBMI = 2.4) who started treatment for overweight and obesity in 48 treatment facilities between July 2005 and October 2006. The facilities offered either inpatient treatment or outpatient programs. Prior to treatment, all participants completed the generic KIDSCREEN-27 HRQOL-questionnaire, the self-perception subscale of the generic KIDSCREEN-52 and the disease-specific obesity module of the KINDL^R^.

The patients' HRQOL was compared to the KIDSCREEN reference sample from the general population by one-way analyses of variance, adjusting for age, gender, and socioeconomic status. Independent t-tests were conducted to compare disease-specific HRQOL scores between patients by gender and age group. Significant mean differences in HRQOL between inpatients and outpatients were explored by one-way analyses of variance, adjusting for age, gender, and zBMI. Effect sizes 'd' were calculated employing the estimated marginal means and the pooled standard deviation (m_treatment _- m_norm_/SD_pooled_).

**Results:**

The patients' HRQOL scores were impaired relative to German norms, with effect sizes up to d = 1.12. The pattern of impairment was similar in boys and girls as well as in children and adolescents. In each of the analyses, at least three of six KIDSCREEN subscales were affected. Regardless of gender and age group, the highest impairments were found in self-perception and physical well-being. Because of the strong decrease in HRQOL in the general population during adolescence, compared to age-specific norms, adolescents were less impaired than were children. However, overweight and obese adolescents (especially females) reported the lowest absolute HRQOL scores. HRQOL varied with the intensity of treatment. Inpatients had significantly lower scores than did outpatients, even after adjusting for age, gender and zBMI.

**Conclusions:**

The results suggest the presence of differences in HRQOL with regard to gender, age, and treatment modality in treatment-seeking overweight and obese youths. Research and clinical practice must consider the particular impairments of inpatients as well as the impairments of (especially female) adolescents.

## Background

Pediatric obesity is a serious public health problem: prevalence rates are currently greater than 20% in the Americas and Europe and are increasing [1,2]. In Germany, 8.7% of 3- to 17-year-olds are overweight and 6.3% are obese [3].

Although overweight and obesity are associated with many severe medical consequences even at a young age [4,5], the most common short-term consequences of pediatric obesity are psychosocial in nature [6,7], such as psychological problems [8], discrimination or teasing [9,10].

Because pediatric obesity can lead to such problems, efforts to examine health-related quality of life (HRQOL) in overweight and obese youths have increased. The multidimensional concept of HRQOL expands the view on health beyond somatic indicators to include the patients' subjective perspective on the physical, psychological, social, and functional aspects of health [11].

Thus far, studies have reported different patterns and magnitudes of impairment with respect to the HRQOL in overweight and obese youths. Studies of obese pediatric patients found considerably reduced HRQOL, primarily in the psychosocial and social functioning domains [12,13]. However, other studies have found significantly reduced HRQOL in psychosocial as well as physical domains [14] or in the physical dimension alone [15]. It is unknown how participant characteristics, such as age range and gender ratio, may account for discrepancies in the patterns of impairment described above. Because HRQOL in young populations differ systematically depending on age and gender [16], research on HRQOL in relation to pediatric obesity must consider these factors.

Another important consideration is the treatment status of those being studied. Impaired HRQOL is more likely in treatment-seeking individuals compared to community-based samples [17,18]. Beyond the role of treatment-seeking status, research in adults has shown that disease-specific HRQOL also varies among obese subgroups of different treatment intensities [19]. However, because treatment status and treatment intensity of overweight and obese youths are highly dependent on their parents, this relationship may be different in a pediatric population. Nevertheless, thus far, no systematic research has attempted to answer this question.

The present paper adds to the existing body of research by systematically analyzing differences between genders as well as differences between age groups regarding obesity-associated impairment of HRQOL. Our analyses focus on whether the extent and the pattern of reductions in HRQOL differ in (1) boys and girls and (2) children and adolescents presenting for obesity treatment. Furthermore, this paper analyzes differences in generic and disease-specific HRQOL between obese pediatric inpatients and outpatients. Therefore, our study aims to examine the differences among pediatric obese patients and to identify patient groups that may be particularly at risk of impaired HRQOL and need special attention.

## Methods

### Participants and Procedures

Patients: The study sample consisted of 1,916 overweight and obese children and adolescents, aged 8 to 16 years, seeking treatment between July 2005 and October 2006 in 48 treatment facilities in Germany. Providers were chosen randomly from all pediatric overweight and obesity treatment facilities in Germany (n = 480) that agreed to participate in the study (n = 135). The providers offered either inpatient treatment or outpatient programs. All programs addressed exercise, diet, and psychosocial aspects. Inpatient treatment is regarded as the most intensive intervention, whereas outpatient programs are considered as less intense.

Upon their admission to the program, the patients' weight and height were recorded. Participants were asked to complete a questionnaire that included demographic data (age, gender, socioeconomic status) as well as instruments measuring HRQOL, such as the KIDSCREEN-27 [20], the self-perception scale of the KIDSCREEN-52 [20,21] and the disease-specific obesity module of the KINDL^R ^[22,23]. The questionnaire was completed prior to treatment. All patients and their parents gave their informed consent to participate. The study was approved by the Ethics Committee of the University of Ulm, Germany.

Reference population: The generic HRQOL-scores of the patients were compared to the representative German KIDSCREEN normal data sample from the German national KIDSCREEN survey. This data sample included 1,494 children and adolescents, aged 8 to 16, in the general population [20]. Sampling was conducted by phone through a random-digital-dialing system. A short standardized interview was used to identify families with children between 8 and 18 years of age. The 'next birthday method' was employed to identify the child to be included in the survey. In Germany, 2430 out of the 4642 contacted families (52.3%) consented to complete a postal KIDSCREEN survey questionnaire. Then, 1873 out of 2413 families (77.6%) with valid addresses returned the completed questionnaires [20]. To facilitate the comparability among the samples, adolescents aged 17 and 18 were excluded from the reference sample.

### Assessment instruments

#### Body Mass Index (BMI) and zBMI

Medical staff recorded the weight and height of patients upon admission. BMI was calculated (kg/m^2^). Standardized BMI (zBMI) was calculated using the LMS method [24], employing national age- and gender-specific values [25]. Overweight and obesity were defined as having a BMI above the age- and sex-specific 90^th ^percentile or 97^th ^percentile, respectively [25].

#### Socioeconomic status (SES)

The family affluence scale (FAS) was employed to measure familial SES in an age-appropriate way [26,27]. The scale includes indicators of the family's material wealth, such as the number of cars and computers, the child having his or her own bedroom, and the number of family holidays in the past 12 months. Depending on responses, children were assigned to three groups (low, intermediate, and high FAS level). Findings from the cross-national Health Behavior in School-aged Children Survey confirmed that the FAS is a valid measure of children's and adolescents' material circumstances [27].

#### KIDSCREEN-27

The KIDSCREEN-27 is a generic HRQOL questionnaire used to assess youths between 8 and 18 years of age. It was developed and validated in a cross-national approach [20,21]. Its five subscales (physical well-being, psychological well-being, autonomy and parents, social support and peers, school environment) offer detailed profile information. The KIDSCREEN-27 self-report version had been shown to have robust psychometric properties. The internal consistency of the subscales was between 0.81 and 0.84, and the test-retest reliability of the subscales ranged from 0.61 to 0.74 [20]. The uni-dimensional KIDSCREEN-10 index was developed from the KIDSCREEN-27 by means of a a Rasch analysis and provides a global HRQOL score. All KIDSCREEN scores are reported as t-values, with higher scores indicating higher HRQOL.

#### KIDSCREEN-52 Self-perception dimension

The self-perception subscale (five items) of the KIDSCREEN-52 reflects the security and satisfaction of youths with themselves and their appearances as well as the value assigned to themselves. The scale had an internal consistency of 0.79 and a test-retest reliability of 0.69 [20].

#### KINDL^R ^obesity module

The disease-specific KINDL^R ^obesity module [22] captures specific experiences associated with pediatric overweight or obesity (shown in additional file 1). This clinical subscale of the generic KINDL^R ^[22,23] includes 12 items referring to six domains: physical well-being, emotional well-being, self-esteem, family, friends, and functional aspects. The KINDL^R ^obesity module was developed for children and adolescents and showed satisfactory internal consistency (Cronbach's alpha = 0.77). The scores were transformed to values between 0 and 100, with higher values indicating better HRQOL.

### Statistical analysis

HRQOL in the treatment-seeking overweight and obese children was compared to that of the reference sample by one-way analyses of variance. Because a comparison between the patients' HRQOL-scores and the German reference would be biased by the higher percentage of girls, older children, and children with lower SES in the group seeking treatment (all three factors are associated with a lower HRQOL), the estimated marginal means of the KIDSCREEN scales were adjusted for age, gender, and SES. We report the results separately for girls and boys as well as for children (ages 8 to 11) and adolescents (ages 12 to 16). Effect sizes 'd' were calculated employing the estimated marginal means (m_treatment _- m_norm_/SD_pooled_) and were interpreted as small (0.20 - 0.50), moderate (0.51 - 0.80) or large (> 0.80) [28]. Independent sample t-tests were conducted to compare disease-specific HRQOL scores between patients by gender and age group. Significant mean differences in HRQOL between inpatients and outpatients were explored using one-way analyses of variance. Univariate generalized linear models were employed separately for each KIDSCREEN scale and the KINDL^R ^obesity module to determine the estimated marginal means, adjusted for age, gender, and zBMI. Again, effect sizes 'd' were calculated employing the estimated marginal means and the pooled standard deviation. All analyses were performed using SPSS (version 15.0 for Windows; SPSS, Inc., Chicago, IL).

## Results

### Sample Characteristics

The treatment-seeking group consisted of 1,916 patients with a mean age of 12.6 years (SD = 2.2; range 8.00 to 16.97 years). Two hundred seventy-two participants (14.2%) were overweight, and 1,644 (85.8%) were obese. The mean zBMI was 2.4 (SD = 0.5). On average, the girls were slightly (0.2 years) but significantly older (t = 2.2; df = 1822.9, p = .029) and had a higher zBMI than did the boys (t = 3.8; df = 1889.3; p < .001). Adolescents (age 12 and older) had a higher zBMI than did children (age 8 to 11) (t = 8.6; df = 1866.1; p < .001). Participants receiving inpatient treatment had significantly higher zBMI scores (t = 10.8; df = 1777.1; p < .001) and were significantly older (t = 18.4; df = 1889.8; p < .001) than the outpatients. A larger percentage of the patients reported lower family affluence (23.1% low, 44.0% medium, and 32.9% high FAS) compared to children from the general population (11.3% low, 47.3% medium, and 41.4% high FAS). Because 12 children in the reference sample (<1%) and 86 children in the treatment-seeking group (4.5%) failed to provide complete data on family affluence, they were excluded from the analyses that adjusted for socioeconomic status. The demographic characteristics are presented in Table 1.

**Table 1:** Sample demographics

	N	Gender n (%)	Age m (SD)	zBMI^a^ m (SD)
**Inpatients**	**871**			
Girls		510 (58.6%)	13.5 (2.0)	2.6 (0.6)
Boys		361 (41.4%)	13.5 (1.8)	2.5 (0.5)
**Outpatients**	**1045**			
Girls		577 (56.2%)	11.9 (2.1)	2.3 (0.5)
Boys		468 (44.8%)	11.7 (1.9)	2.3 (0.4)
**Children **(aged 8 to 11)	**775**			
Girls		427 (55.1%)	10.4 (1.1)	2.3 (0.5)
Boys		348 (44.9%)	10.4 (1.1)	2.3 (0.4)
**Adolescents **(aged 12 to 16)	**1141**			
Girls		660 (57.8%)	14.1 (1.3)	2.6 (0.6)
Boys		481 (42.2%)	13.9 (1.3)	2.4 (0.5)
**Total**	**1916**			
Girls		1087 (56.7%)	12.7 (2.2)	2.5 (0.5)
Boys		829 (43.3%)	12.5 (2.1)	2.4 (0.5)

^a ^Standardized body mass index (BMI) calculated by the LMS method [24]

### Generic HRQOL in children and adolescents with overweight and obesity

The HRQOL of the patients was impaired compared to the girls (Table 2) and boys (Table 3) in both age groups in the reference sample. Regardless of gender and age, a reduced HRQOL was found in at least three subscales: physical well-being, psychological well-being, and self-perception. The highest impairment was always observed in self-perception and physical well-being, with effect sizes ranging from d = 0.94 to d = 1.12. Because the decline of the patients' absolute HRQOL scores between childhood and adolescence corresponded to the decline observed in the general population, some effect sizes of impairment were smaller in adolescents than in children (Table 2, Table 3) (additional file 2).

**Table 2:** Comparison of HRQOL in treatment-seeking overweight and obese girls and the KIDSCREEN norm sample^a^

	Girls 8 - 11					Girls 12 - 16				
KIDSCREEN-scale	Norm (n = 295) **m **(SE)	Patients (n = 427) m (SE)	F	p	d	Norm (n = 465) m (SE)	Patients (n = 660) m (SE)	F	p	d
Physical Well-being	**54.9 **(0.5)	**45.4 **(0.4)	200.5	<.001	0.94	**50.4 **(0.4)	**41.3 **(0.3)	321.8	<.001	0.98
Psychol. Well-being	**55.5 **(0.5)	**48.4 **(0.5)	102.2	<.001	0.72	**49.7 **(0.4)	**44.5 **(0.4)	86.0	<.001	0.56
Parents	**53.8 **(0.6)	**51.1 **(0.5)	14.2	<.001	0.29	**51.4 **(0.4)	**50.2 **(0.4)	4.6	0.03	0.14
Peers	**51.5 **(0.6)	**50.1 **(0.5)	3.0	0.08	ns^b^	**50.7 **(0.5)	**50.1 **(0.4)	1.1	0.31	ns^b^
School	**56.0 **(0.6)	**54.5 **(0.5)	3.8	0.05	ns^b^	**50.1 **(0.4)	**49.7 **(0.4)	0.7	0.41	ns^b^
KIDSCREEN-Index	**55.8 **(0.6)	**50.4 **(0.5)	50.2	<.001	0.53	**49.8 **(0.4)	**46.7 **(0.3)	35.9	<.001	0.37
Self-perception	**54.7 **(0.5)	**42.8 **(0.4)	338.1	<.001	1.12	**47.0 **(0.4)	**38.0 **(0.3)	344.4	<.001	1.01

^a ^Adjusted for age, gender, and socioeconomic status

^b ^ns = not significant; only significant effects sizes are reported

**Table 3:** Comparison of HRQOL in treatment-seeking overweight and obese boys and the KIDSCREEN norm sample^a^

	Boys 8 - 11					Boys 12 - 16				
KIDSCREEN-scale	Norm (n = 305) m (SE)	Patients (n = 348) m (SE)	F	p	d	Norm (n = 419) m (SE)	Patients (n = 481) m (SE)	F	p	d
Physical Well-being	**55.7 **(0.5)	**46.4 **(0.5)	195.2	<.001	0.96	**52.8 **(0.4)	**42.8 **(0.4)	293.3	<.001	1.04
Psychol. Well-being	**55.8 **(0.5)	**49.9 **(0.5)	59.7	<.001	0.58	**52.7 **(0.4)	**47.5 **(0.4)	69.7	<.001	0.57
Parents	**52.4 **(0.5)	**49.9 **(0.5)	10.9	<.001	0.27	**51.8 **(0.4)	**51.5 **(0.4)	0.2	0.62	ns^b^
Peers	**50.3 **(0.6)	**49.1 **(0.6)	2.3_)_	0.13	ns^b^	**49.3 **(0.5)	**49.1 **(0.5)	0.6	0.80	ns^b^
School	**53.1 **(0.6)	**52.0 **(0.6)	2.1	0.15	ns^b^	**49.8 **(0.5)	**49.3 **(0.4)	0.5	0.48	ns^b^
KIDSCREEN-Index	**54.9 **(0.5)	**49.9 **(0.5)	43.4	<.001	0.51	**51.8 **(0.4)	**49.0 **(0.4)	23.5	<.001	0.34
Self-perception	**57.3 **(0.5)	**45.3 **(0.5)	294.2	<.001	1.10	**52.0 **(0.4)	**42.3 **(0.4)	310.8	<.001	1.06

^a ^Adjusted for age, gender, and socioeconomic status

^b ^ns = not significant; only significant effects sizes are reported

### Disease-specific HRQOL in overweight and obese children and adolescents

In male patients, comparable disease-specific HRQOL scores were observed in children and adolescents (m = 66.0, SD = 17.1 versus m = 67.3, SD = 16.5). However, female adolescent patients reported significantly lower disease-specific HRQOL than did female children (m = 61.5, SD = 17.8 versus m = 64.4, SD = 18.2; t = 2.6; df = 1058; p = .010, d = 0.16). Thus, no significant gender differences in disease-specific HRQOL were observed during childhood. However, in adolescence, female patients reported significantly lower disease-specific HRQOL than their male counterparts (t = 5.6; df = 1106; p < .001, d = 0.34).

### Differences in generic and disease-specific HRQOL across treatment modalities

Table 4 displays the differences in HRQOL between patients with different treatment intensities (inpatients versus outpatients). Compared to the outpatient participants, inpatient participants reported significantly lower HRQOL scores in physical well-being, psychological well-being, school, self-perception, the overall HRQOL index, and disease-specific HRQOL. After adjusting for age, gender and zBMI, these significant differences remained in all subscales. However, after adjusting for covariates, the differences were less pronounced and effect sizes were reduced by approximately half.

**Table 4:** Generic and disease-specific HRQOL by treatment modality

	Total (n = 1916)	Inpatients (n = 871)	Outpatients (n = 1045)	F	p^c^	d
**KIDSCREEN-subscale**	m (SE)	m (SE)	m (SE)			
**Physical Well-being**						
Unadjusted^a^	43.5 (0.2)	41.3 (0.3)	45.3 (0.3)	98.3	<.001	0.46
Adjusted^b^		42.4 (0.3)	44.4 (0.3)	22.1	<.001	0.23
**Psychological Well-being**						
Unadjusted^a^	47.1 (0.2)	45.4 (0.3)	48.4 (0.3)	48.5	<.001	0.32
Adjusted^b^		46.2 (0.3)	47.7 (0.3)	10.7	<.001	0.16
**Parents**						
Unadjusted^a^	50.6 (0.3)	50.9 (0.3)	50.3 (0.3)	1.9	0.17	ns^c^
Adjusted^b^		50.9 (0.3)	50.2 (0.3)	2.2	0.14	ns^c^
**Peers**						
Unadjusted^a^	49.6 (0.3)	49.5 (0.4)	49.7 (0.4)	0.2	0.69	ns^c^
Adjusted^b^		49.5 (0.4)	49.7 (0.4)	0.04	0.82	ns^c^
**School**						
Unadjusted^a^	51.0 (0.2)	49.4 (0.3)	52.4 (0.3)	42.1	<.001	0.30
Adjusted^b^		50.1 (0.4)	51.8 (0.3)	11.4	<.001	0.17
**KIDSCREEN-Index**						
Unadjusted^a^	48.6 (0.2)	47.3 (0.3)	49.7 (0.3)	35.0	<.001	0.28
Adjusted^b^		48.0 (0.3)	49.2 (0.3)	7.8	0.01	0.14
**Self-perception**						
Unadjusted^a^	41.4 (0.2)	39.8 (0.3)	42.7 (0.2)	70.3	<.001	0.39
Adjusted^b^		40.9 (0.3)	41.8 (0.2)	6.7	0.01	0.13
**KINDL^R ^obesity module**						
Unadjusted^a^	64.4 (0.4)	60.6 (0.6)	67.5 (0.5)	74.5	<.001	0.39
Adjusted^b^		61.3 (0.6)	66.9 (0.5)	42.7	<.001	0.32

^a ^Cell entries represent unadjusted mean and standard error

^b ^Estimated marginal means adjusted for age, gender and zBMI

^c ^ns = not significant; only significant effects sizes are reported

## Discussion

Our findings confirm previous research reports on the considerably reduced HRQOL in overweight and obese pediatric patients, such as the marked impairment of physical well-being [12-15,17]. Furthermore, overweight and obese children and adolescents reported impaired self-perception and psychological well-being, suggesting the importance of very seriously considering the psychosocial correlates of their condition. Even impairments in the parent dimension - especially in younger patients - were observed. Interestingly, no impairment in the KIDSCREEN peers dimension was reported. In contrast to previous research [12-14,17] that administered the PedsQL [29], which is very sensitive to problems such as prejudice and stigmatization (*'Other kids do not want to be my friend' *or *'Other kids tease me'*), the KIDSCREEN provides information on how the child experiences the friendships that he or she has in terms of the quality of interaction and perceived support (*'Have you had fun with your friends?', 'Were you able to rely on your friends?'*). In our study, the quality of peer relations was hardly reduced, although our patient sample was likely to experience an increased level of peer rejection [9,30].

Consistent with previous findings [12,15,17], the pattern of impairment in HRQOL was very similar in both genders. In boys and girls, the same dimensions were impaired to almost equal extents according to the effect sizes. The only exceptions were that young girls experienced slightly higher emotional impairment than did boys and that among adolescents, only girls reported reduced scores in the parent dimension. Although adolescent girls had lower scores than their male counterparts, these findings are not unique to overweight and obese females. The differences correspond to the lower HRQOL adolescent girls experience in general [16] and support the finding that overweight does not operate differently among males and females [18].

Speculations that gender differences in obesity-related impairments of HRQOL are small in children but increase in adolescents [15] were not supported by our data in regard to generic HRQOL. The observed increase in HRQOL differences between male and female patients at the onset of puberty corresponds to observations in community samples [16]. However, regarding the disease-specific HRQOL, gender differences emerged in adolescence, with girls being more negatively affected by their condition.

The pattern of obesity-related impairments in the different HRQOL subdimensions was also very similar in children and adolescents. However, determining the age group with the greatest obesity-associated impairment may be explored in different ways. Tables 2 and 3 display that, compared to age-specific norms, overweight and obese children experienced higher impairment in several HRQOL dimensions than did adolescents. Nevertheless, the absolute mean scores reveal that overweight and obese adolescents reported the lowest HRQOL. Thus, relative to normal-weight youths of the same age, obese children experienced higher impairment. With regard to the absolute scores, adolescents were the most impaired. The relatively lower impairment in adolescent patients, despite the decrease of HRQOL in almost every subscale after puberty, resulted from the larger reduction of HRQOL during adolescence in the reference population. Because adolescents in general experience a strong decline in HRQOL, the scores of the patient sample converge to the healthy adolescents' scores.

Our results suggest that practitioners must pay attention to the extremely low HRQOL in adolescent patients, particularly the girls. They have exceptionally low HRQOL scores, and special consideration must be paid in providing them with the appropriate treatment. However, our data also suggest the necessity of focusing early on children's obesity-associated impairments of HRQOL.

This study shows that HRQOL varies between clinical samples with different intensities of treatment. Inpatients had lower disease-specific and generic HRQOL with regard to their physical and psychological well-being, school functioning, and self-perception. The results of the adjusted analyses indicated that the extent of these differences might be partly attributed to the special characteristics of the inpatient population, such as age, zBMI and gender. However, even in the adjusted comparison, inpatients had a higher level of impairment in their physical and psychological well-being, school-related well-being, self-perception, and disease-specific HRQOL.

These results may not only sensitize practitioners to their inpatients as a special treatment group but may also have implications for the interpretation of research results regarding HRQOL and pediatric obesity. It has been questioned whether the results on HRQOL from treatment samples may be transferred to community samples [14,17,31]. By pointing out differences between inpatients and outpatients, our study further shows that the results from special treatment populations cannot even be transferred to other treatment populations.

In clinical practice, low HRQOL might be addressed by diverse measures, such as a standardized assessment of psychological aspects when patients present for treatment, and by an extension of special therapy modules, e.g., focusing self-perception. Furthermore, it might be helpful to assess HRQOL for evaluating treatment options and determining whether pediatric obese patients may benefit from specific therapies.

Beyond the implications for research and treatment, our findings also point toward the responsibility of the social environment. The strong impairments in self-perception and psychological well-being can be consequences of stigmatization and teasing experiences [9,32,33]. Thus, improved attitudes towards overweight and obese individuals in society might be an important component for the improvement of HRQOL in affected individuals.

The strengths of the present study include its large, geographically diverse sample from 48 treatment facilities in Germany. Furthermore, important predictors of HRQOL such as age, gender and SES were considered when comparing the patient sample to the norm. The current study includes, for the first time, a systematic examination of the role of treatment intensity and the disease-specific and generic HRQOL in pediatric overweight and obese patients. Another strength of the study lies in the use of an HRQOL instrument that allows a cross-cultural comparison of measurements. With respect to the global dimension of pediatric obesity, this cross-culturally comparable measurement supports the growing knowledge in this field. Furthermore, because the height and weight were measured by medical staff, the BMI data are highly reliable. A limitation in this study is the lack of differentiation among inpatient and outpatient programs. Additionally, our findings that showed a higher impairment in inpatients may be limited to Germany. In particular, because access to treatment and access to diverse treatment modalities are highly dependent on a country's health care system, the characteristics of inpatients and outpatients may be different in other countries.

## Conclusions

Our study shows a considerably reduced HRQOL in overweight and obese pediatric clinical samples. The pattern of impairment in HRQOL was very similar in boys and girls as well as in children and adolescents. Regardless of gender and age, the highest impairments were observed in self-perception and physical well-being. Nevertheless, the results from our study suggest that differences in HRQOL across gender, age groups, and treatment modalities have to be taken into account to address the correlates of this condition appropriately. Inpatients were identified as a considerably impaired treatment group. In addition, because they have extremely low HRQOL scores, female adolescents should receive particular attention during treatment. However, putting into perspective the noticeable impairment of the patients' HRQOL regarding age- and gender-specific norms, it should be acknowledged that children of both genders are already affected.

With respect to previous and further research on HRQOL in pediatric obese patients, our results implicate that interpretations of all research findings need to consider the specific characteristics of the underlying treatment population.

## Competing interests

The authors declare that they have no competing interests.

## Authors' contributions

NW performed the statistical analyses and interpretation and drafted the manuscript. MB, RH, UH, RM, CG, TR, JW, AEF, and URS made substantive contributions to the conception and design of the study, organized and conducted the study, and critically revised the manuscript for important intellectual content. All authors read and approved the final manuscript.

## Supplementary Material

Additional file 1 **KINDLR_ObesityModule**. This additional file includes the disease-specific HRQOL questionnaire (KINDL^R ^obesity module). Click here for file

Additional file 2 **Figures**. This additional file includes figures displaying generic and disease-specific HRQOL of children and adolescents in the reference population vs. the overweight and obese patients. They illustrate the information presented in Tables 2 and 3. Click here for file
