# Supplementary material for: Health-related quality of life in overweight and obese youths: Results of a multicenter study
Source: Health Qual Life Outcomes. 2010 Apr 7;8:36. doi: 10.1186/1477-7525-8-36 (PMC2868813; doi:10.1186/1477-7525-8-36)
Supplement: Additional file 1 — KINDLR_ObesityModule. This additional file includes the disease-specific HRQOL questionnaire (KINDLR obesity module). [file 1477-7525-8-36-S1.PDF]

Are you overweight (obese)?

☐ **Yes** ☐ **No**

During the past week…	never	seldom	some-times	often	all the time
1. ... I felt fat and immobile	☐	☐	☐	☐	☐
2. ... I got out of breath quickly and I was puffed out quickly	☐	☐	☐	☐	☐
3. ... I was sad and depressed because of my weight	☐	☐	☐	☐	☐
4. ... I was annoyed by my many attempts at getting thinner	☐	☐	☐	☐	☐
5. ... I felt ashamed because of my weight	☐	☐	☐	☐	☐
6. ... I was dissatisfied with myself because of my weight	☐	☐	☐	☐	☐
7. ... my family grumbled at me because of my weight	☐	☐	☐	☐	☐
8. ... I had to keep an eye on my weight during meals at home	☐	☐	☐	☐	☐
9. ... I was teased by others because of my weight	☐	☐	☐	☐	☐
10. ... I was left out by others when they did things together, because of my weight	☐	☐	☐	☐	☐
11. ... I was distracted during lessons by the thought of food	☐	☐	☐	☐	☐
12. ... I was able to take part well in sport at school, in spite of my weight	☐	☐	☐	☐	☐

13. How often during the past week did you have complaints because of being overweight (obese)?

☐ never ☐ seldom ☐ sometimes ☐ often ☐ all the time

14. How severe were your complaints because of being overweight during the past week?

☐ none at all ☐ somewhat severe ☐ moderately severe ☐ fairly severe ☐ very severe

15. How much did it bother you being overweight during the past week?

☐ not at all ☐ somewhat ☐ moderately ☐ fairly much ☐ very much
