# Supplementary material for: Health-related quality of life in overweight and obese youths: Results of a multicenter study
Source: Health Qual Life Outcomes. 2010 Apr 7;8:36. doi: 10.1186/1477-7525-8-36 (PMC2868813; doi:10.1186/1477-7525-8-36)
Supplement: Additional file 2 — Figures. This additional file includes figures displaying generic and disease-specific HRQOL of children and adolescents in the reference population vs. the overweight and obese patients. They illustrate the information presented in Tables 2 and 3. [file 1477-7525-8-36-S2.PDF]

Generic and disease-specific HRQOL (KIDSCREEN-27 and KINDL[R] obesity module) of children and adolescents in the reference population vs. in overweight and obese patients

Figure 1a. Physical Well-being

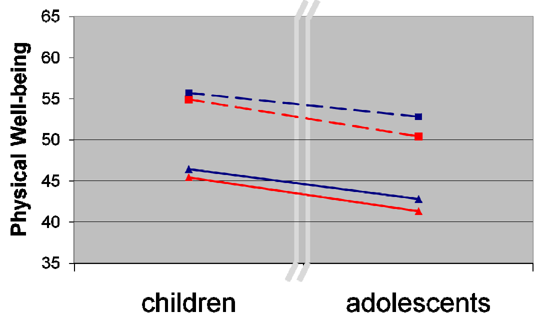

Figure 1b. Psychological Well-being

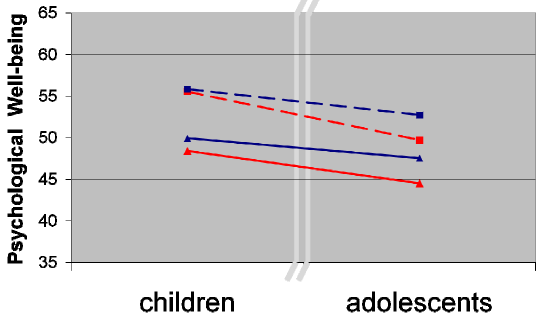

Figure 1c. Parents

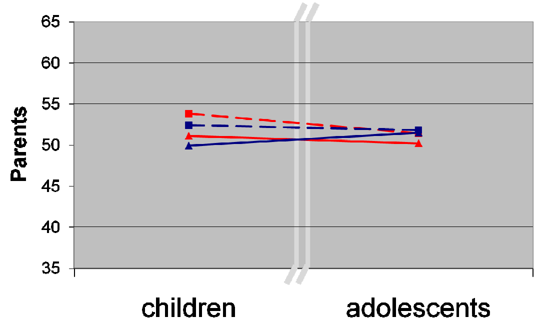

Figure 1d. Peers

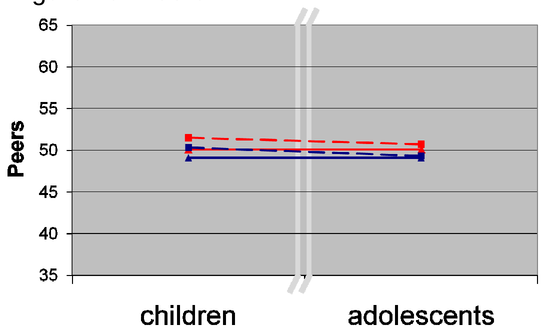

Figure 1e. School

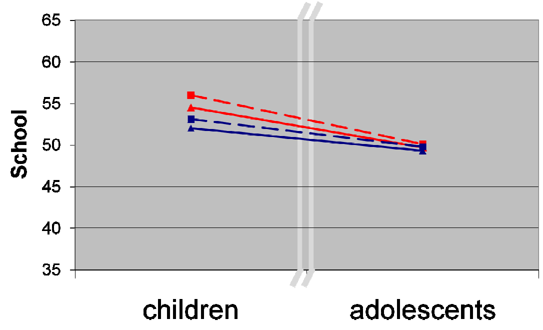

Figure 1f. KIDSCREEN-10 Index

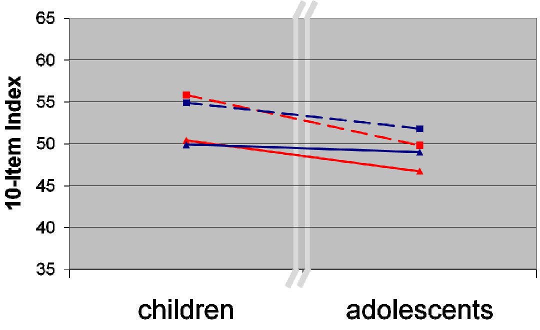

Figure 1g. Self-perception

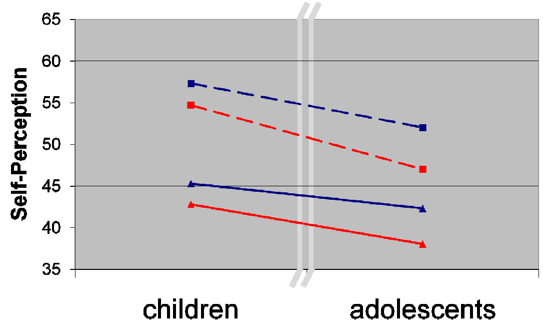

Figure 1h. KINDL[R] obesity module

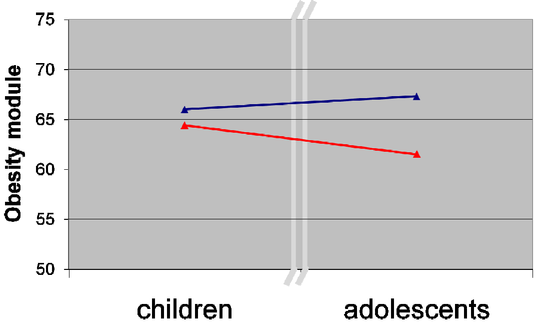

Red permanent line – treatment sample girls
Red interrupted line – norm population girls
Blue permanent line – treatment sample boys
Blue interrupted line – norm population boys
Plumb lines indicate that no "real" trends are shown: children's data and adolescents' data are independent.
